# Fatal Cardiogenic Shock in Patients with Heart Failure: National Mortality Trends, Forecasting, and the Influence of Atherosclerotic Cardiovascular Disease in the United States in 1999–2023

**DOI:** 10.3390/biomedicines14081847

**Published:** 2026-08-17

**Authors:** Faizan Ahmed, Muhammad Abdullah, Arsalan Ahmad Butt, Muhammad Faizan Tahir, Shiraz Aslam, Taha Alam, Haris Bin Tahir, Yusaf Kalson, Muhammad Shees Hunain, Tehmasp Rehman Mirza, Mohamed Bakr, Mohammad Amir Hossain, Fawaz Alenezi

**Affiliations:** 1 Department of Internal Medicine, Jersey Shore University Medical Center, Neptune, NJ 07753, USA; 2 Department of Internal Medicine, Shalamar Medical and Dental College, Lahore 54000, Pakistan; mohd_abdullah2003@outlook.com (M.A.); faizantahir5678@gmail.com (M.F.T.); shirazaslam2002@gmail.com (S.A.); shery.h54321@gmail.com (M.S.H.);; 3 Department of Internal Medicine, Aga Khan University Hospital, Karachi 74800, Pakistan; arsalanbutt2012@gmail.com; 4 Department of Internal Medicine, Ameer-ud-Din Medical College, Lahore 54000, Pakistan; tahaaalam9@gmail.com; 5 Department of Internal Medicine, Lahore General Hospital, Lahore 54000, Pakistan; harristahirchh@gmail.com; 6 School of Medicine, St. George’s University, St. George’s, Grenada; 7 Division of Cardiology, Duke University, Durham, NC 27710, USA

**Keywords:** atherosclerotic heart disease, heart failure, cardiogenic shock, mortality trends, United States

## Abstract

**Background:** Atherosclerotic heart disease (AHD) remains a leading cause of global mortality and a major driver of fatal cardiac syndromes. Ischemic myocardial injury from AHD can lead to ventricular remodeling and myocyte loss, progressing to heart failure (HF) and, in advanced stages, cardiogenic shock (CS). However, long-term national mortality trends for deaths involving the combined burden of AHD, HF, and CS remain insufficiently characterized. **Methods:** Mortality data from 1999 to 2023 were obtained from the CDC WONDER Multiple Cause of Death database. Deaths involving CS (ICD-10 R57.0), HF (I50, I50.1, I50.9), and AHD (I25.1) in adults were identified. Age-adjusted mortality rates (AAMRs) per 1,000,000 population were calculated and stratified by sex, race/ethnicity, and geographic region. Temporal trends were analyzed using Joinpoint regression to estimate annual percent changes (APCs) and average annual percent changes (AAPCs). Autoregressive integrated moving average (ARIMA) modeling was used to forecast mortality trends through 2035. **Results:** A total of 15,631 deaths were attributed to AHD-associated HF and CS during the study period. Overall AAMRs increased significantly (AAPC 3.75%; 95% CI 3.32–4.29; *p* < 0.01). Mortality rates declined between 1999 and 2006 (APC −7.55%; 95% CI −13.15 to −5.23; *p* < 0.01), increased modestly from 2006 to 2013 (APC 3.94%; 95% CI −1.47 to 8.91), and rose sharply from 2013 to 2023 (APC 12.33%; 95% CI 11.27–14.46; *p* < 0.01). Males had higher AAMRs than females in 2023 (9.26 vs. 3.39 per 1,000,000). Non-Hispanic White individuals accounted for the highest number of deaths (11,878), followed by non-Hispanic Black individuals (1708), while non-Hispanic American Indian populations had the lowest counts (39). Regionally, the West (7.31) and South (5.92) exhibited the highest final AAMRs. State-level AAMRs ranged from 2.49 in Minnesota to 8.27 in Nevada. **Conclusions:** Mortality involving AHD, HF, and CS has risen substantially in the United States since 2013, with marked demographic and geographic disparities. These findings highlight a growing burden of advanced ischemic heart disease and heart failure and underscore the need for earlier intervention and improved access to advanced HF therapies to prevent progression to cardiogenic shock and death.

## 1. Introduction

Cardiogenic shock (CS) is a life-threatening cardiac condition characterized by low cardiac output that leads to end-organ hypoperfusion and tissue hypoxia. Common causes of CS include acute myocardial ischemia, heart failure, mechanical complications such as mitral regurgitation, cardiac tamponade, or ventricular wall rupture, and contractility abnormalities including arrhythmias and cardiomyopathy. The diagnosis of CS is confirmed through specific clinical criteria, which include a systolic blood pressure below 90 mm Hg for at least 30 min (with or without mechanical support), a cardiac index of 2.2 L/min/m^2^ or less with support, or below 1.8 L/min/m^2^ without support, and high serum lactate levels [1]. Despite advancements in treatment, CS continues to be the leading cause of death among patients with acute myocardial infarction (AMI) [2].

Heart failure is a common downstream consequence of the ischemic and structural injury described above, and frequently precedes the development of cardiogenic shock. According to the American Heart Association (AHA), heart failure (HF) is a complex clinical syndrome resulting from structural or functional impairment of ventricular filling or the ejection of blood [3]. HF is becoming increasingly prevalent, affecting an estimated 26 million individuals worldwide. In the United States alone, more than 6.7 million adults are currently living with this condition [4].

Atherosclerotic coronary disease is the principal upstream driver of the ischemic injury that leads to both heart failure and cardiogenic shock, making it central to understanding the natural history of these conditions. Atherosclerosis is a vascular disease involving endothelial injury, inflammation, lipid accumulation, and plaque formation within the intimal layer of the arterial wall. It serves as an underlying mechanism for numerous cardiovascular disorders, including coronary artery disease (CAD). Atherosclerotic diseases significantly contribute to the global burden of cardiovascular disease (CVD). According to the 2022 Global Burden of Disease (GBD) study, the global prevalence of CAD was approximately 315 million [5]. In this study, AHD is defined by ICD-10 code I25.1 on the death certificate, reflecting chronic ischemic heart disease rather than an acute coronary event.

Previous research has investigated mortality trends associated with CS in HF [4] and explored deaths related to atherosclerotic heart disease (AHD) [5]. However, mortality trends among patients with HF and AHD who died with CS remain inadequately studied. Given that AHD is a major contributor to cardiovascular morbidity and mortality, addressing this knowledge gap is essential.

This study aims to examine mortality trends among patients who died with cardiogenic shock, heart failure, and atherosclerotic heart disease. The findings may help guide the development of targeted preventive and therapeutic interventions.

## 2. Methodology

### 2.1. Data Source

The Centre for Disease Control and Prevention Wide-ranging Online Data for Epidemiological Research (CDC WONDER) database was used to analyze mortality trends in adults (aged ≥ 25) with cardiogenic shock (CS), heart failure (HF), and atherosclerotic heart disease (AHD) across the United States between 1999 and 2023. Atherosclerotic heart disease was defined as an underlying cause of death per ICD-10 code I25.1, an administrative death-certificate code rather than an angiographic or clinical diagnosis. The International Classification of Diseases, Tenth Revision, (ICD-10) codes R57.0 (cardiogenic shock) and I50 (Heart Failure) were used for the identification of the Multiple Cause of Death records. No exclusion was applied for acute myocardial infarction (I21–I22) or sudden cardiac death (I46) codes co-occurring on the same record. Because the final four years of the study period (2020–2023) overlap with the COVID-19 pandemic, we did not exclude records with a concurrent COVID-19 code (U07.1); this is addressed further in the Limitations. Since the data utilized was publicly available and anonymous, institutional approval was not needed.

### 2.2. Data Extraction

Data was extracted from the CDC WONDER database for all 51 states (District of Columbia included) in the United States of America. The data included demographic information, population counts, year, and geographical location. Demographic information included gender, age, race, and ethnicity. According to the gender specifications on death certifications, adults were categorized into males and females. Pre-specified age groups, set by the database, categorized age into 25–34, 35–44, 45–54, 55–64, 65–74, 75–84, and ≥85 years. As given by CDC WONDER, race and ethnicity were classified into non-Hispanic (NH) White, non-Hispanic Black or African American, Hispanic or Latino, non-Hispanic American Indian or Alaska Native, and Asian or Pacific Islander. The geographical data was grouped into broad categories of Metropolitan (Large Central Metro, Large Fringe Metro, Medium Metro and Small Metro) and Nonmetropolitan (Micropolitan and NonCore). Regions were classified as Northeast, Midwest, South, and West. The CDC WONDER age distribution of deaths over time was characterized as the proportion of deaths occurring in adults aged 65 years and older versus 25–64 years, by 5-year period, rather than as a mean age (Supplemental Table S1).

### 2.3. Statistical Analysis

The age-adjusted mortality rates (AAMRs) per 1,000,000 people were used to assess the nationwide trends in CS, HF, and AHD mortality rates. AAMR was determined by standardizing CS, HF, and AHD deaths to the year-2000 population. The annual percentage change (APC) in age-adjusted mortality rate with a 95% CI was analyzed using the Joinpoint Regression Program (Joinpoint V 5.0, National Cancer Institute) to recognize the significance of the annual patterns. The significant changes in age-adjusted mortality rate for CS, HF, and AHD were determined by fitting long linear regression models and identifying the temporal variations. The APC was said to be increasing or decreasing if the slope representing the change in mortality significantly differed from zero. This was determined using two-tailed *t*-tests. A *p*-value of < 0.05 was considered statistically significant.

Time-series forecasting of mortality trends was performed using autoregressive integrated moving average (ARIMA) models implemented in Python version 3.13.2 with the statsmodels package. Model parameters (p, d, q) were assessed iteratively, with the search range for each parameter increased from orders 0–3 to 0–5 to ensure a more robust model selection; the optimal model was selected based on the lowest root mean square error (RMSE) during validation.

## 3. Results

### 3.1. Overall

**Figure 1:**
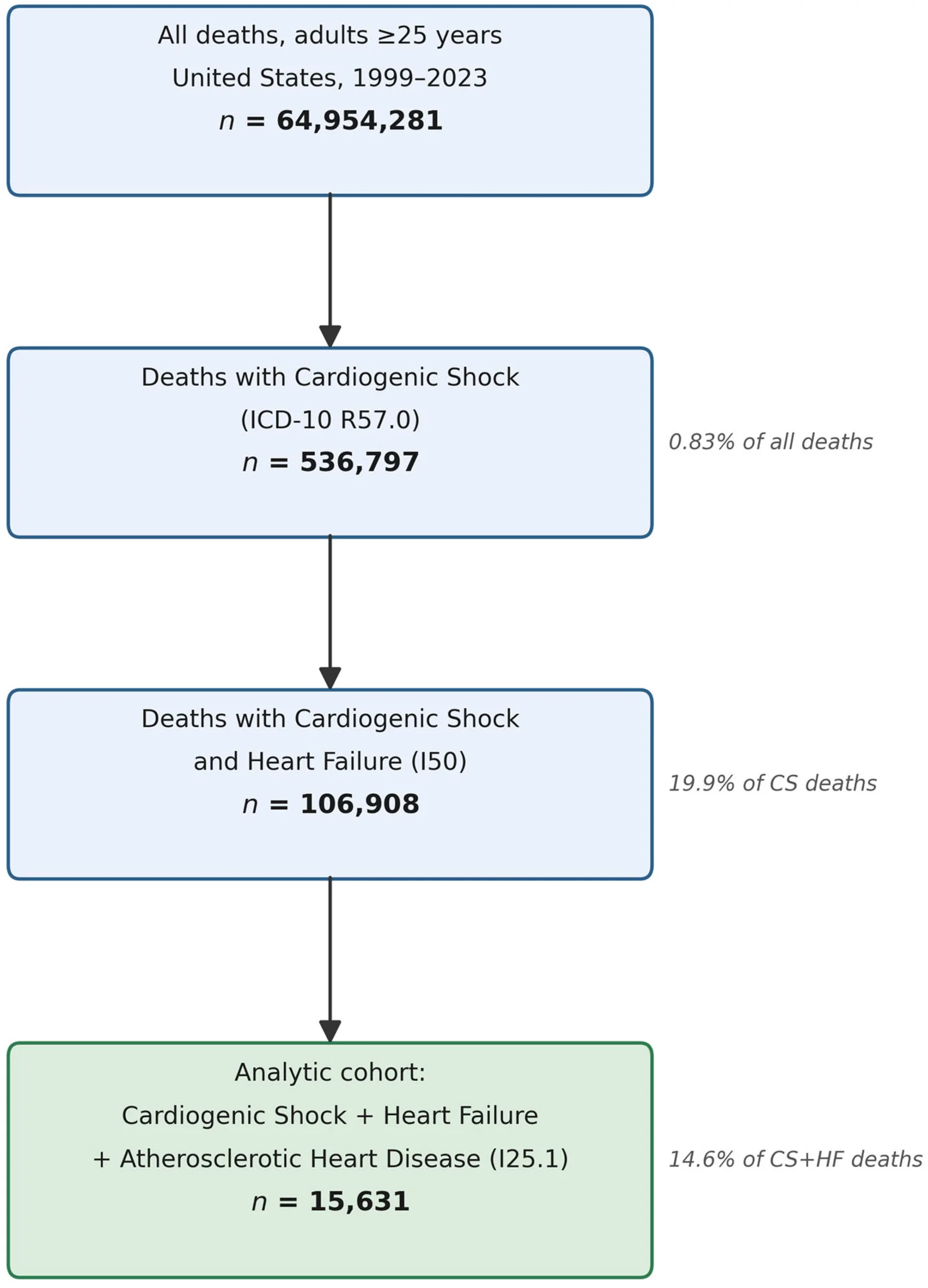
Overall cardiogenic shock, heart failure and atherosclerotic heart disease-related mortality counts.

**Figure 2:**
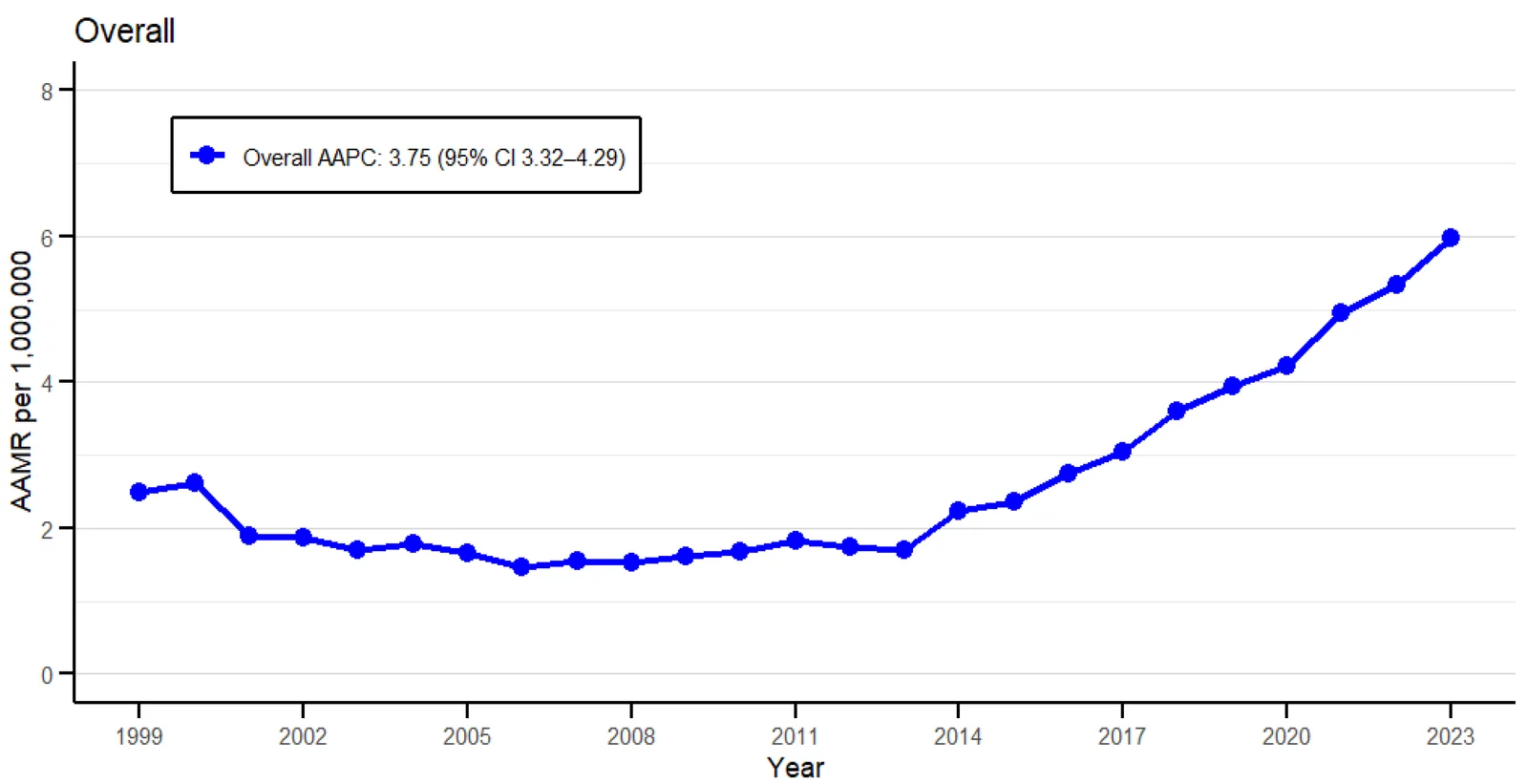
Overall cardiogenic shock, heart failure and atherosclerotic heart disease-related age-adjusted mortality rate (AAMR) per 1,000,000 in the United States, from 1999 to 2023.

Between 1999 and 2023, a total of 15,631 deaths were caused by cardiogenic shock (CS), heart failure (HF), and atherosclerotic heart disease (AHD) in the United States (Figure 1). The age-adjusted mortality rates (AAMRs) per million showed an increase from 2.50 (95% CI 2.26–2.73) in 1999 to a peak of 5.97 (95% CI 5.68–6.26) in 2023 (Figure 2). Mortality rates demonstrated a marked upward trend over the study period, with an average annual percentage change (AAPC) of 3.75* (95% CI 3.32–4.29, *p* < 0.000001). With an APC of 12.33% (95% CI 11.27–14.46, *p* < 0.000001) the greatest upsurge was noted between 2013 and 2023 (Supplemental Tables S2–S4).

### 3.2. Gender

**Figure 3:**
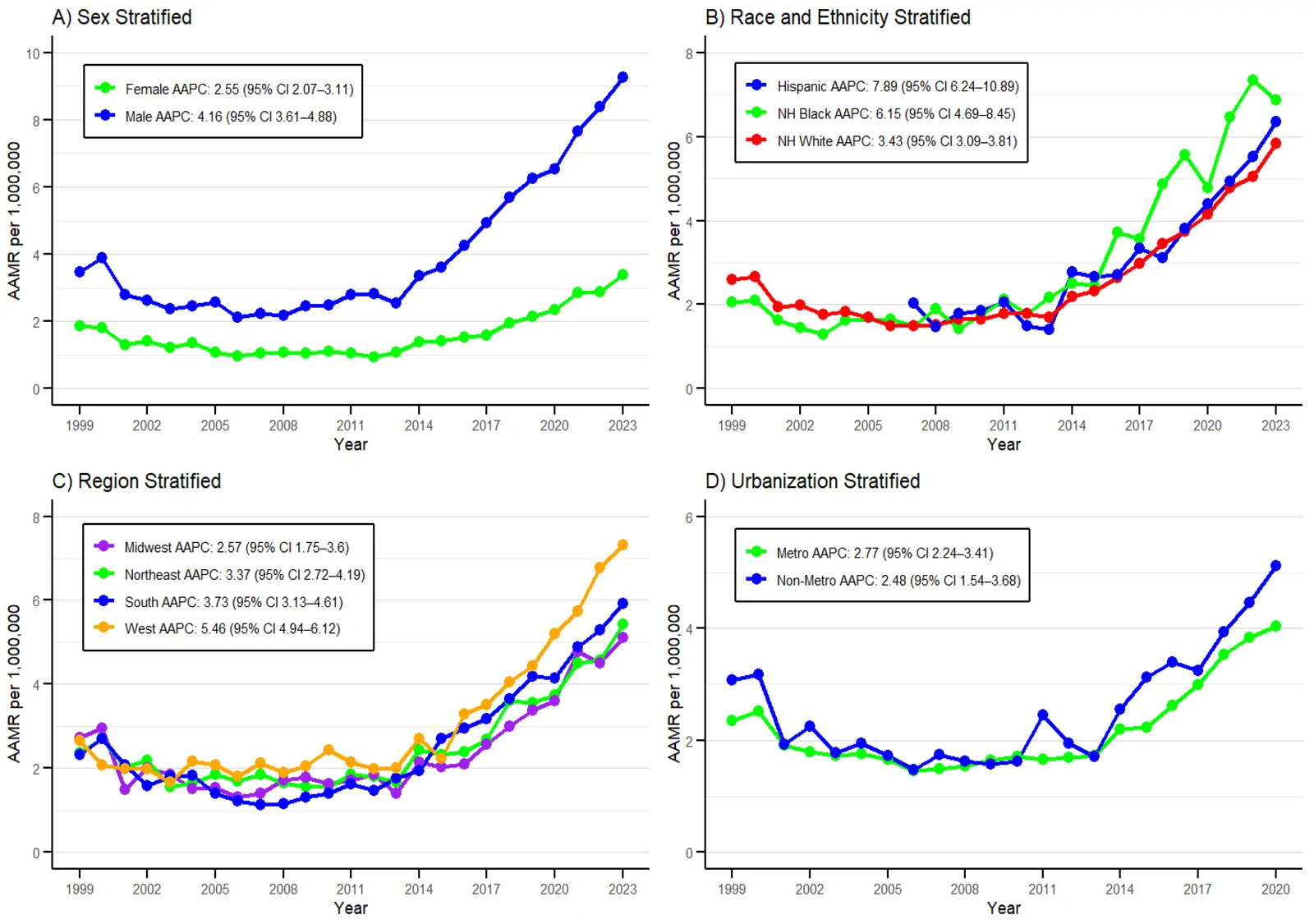
Cardiogenic shock, heart failure and atherosclerotic heart disease-related age-adjusted mortality rates (AAMRs) per 1,000,000 in the United States, 1999 to 2023, stratified by sex, race, region and urbanization status.

Mortality trends stratified by gender exhibited persistently higher AAMRs among males compared to females (Figure 3A). From 1999 to 2023, Males accounted for 10,231 deaths, almost double of that of females, with 5400. Both male and female populations showed increases in mortality trends from 1999 to 2023, with their AAMRs peaking in 2023 (9.26 vs. 3.39). Males saw their highest increase between 2013 and 2023 with an APC of 12.42* (95% CI 11.16–15.85, *p* < 0.000001), while females observed their greatest difference between 2012 and 2023 with an APC of 11.39* (95% CI 10.09–14.08, *p* < 0.000001). The overall trend for males increased with an AAPC of 4.16* (95% CI 3.60–4.88, *p* < 0.000001), while females showed a slower rise with an AAPC of 2.55* (95% CI 2.07–3.11, *p* < 0.000001). (Supplemental Tables S3 and S4).

### 3.3. Race/Ethnicity

The database for race was divided into two main categories: HISPANIC or LATINO and NON-HISPANIC ORIGIN, which included four races (American Indian or Alaska Native, Asian or Pacific Islander, Black or African American, and White) (Figure 3B). Between 1999 and 2023, among non-Hispanic groups, the highest average AAMR was observed among Black or African Americans at 3.02. peaking in 2022 at 7.34 (95% CI 6.29–8.38). This was followed by White groups, who averaged at an AAMR of 2.58 peaking in 2023 at 5.84. Comparing these two races, from 1999 to 2023, mortality rates rose in both, with the higher AAPC observed in Black or African Americans (6.15*, 95% CI 4.69–8.45, *p* < 0.000001) compared to White (3.43*, 95% CI 3.09–3.81, *p* < 0.000001) groups. Black or African American populations showed their steepest rise between 2009 and 2023 with an APC of 12.41% (95% CI 10.30–18.64, *p* < 0.000001), while the White population showed the greatest rise between 2013 and 2023 with an APC of 12.14% (95% CI 11.24–13.41, *p* < 0.000001). The Non-Hispanic, American Indian or Alaska Native and Asian or Pacific Islander and the Hispanic or Latino races showed suppressed results (Supplemental Tables S3 and S5).

### 3.4. Age Group

The mortality trends were divided into two age groups, adults (25–64 years) and the elderly (65+ years). For the adults, 2713 deaths were recorded between 1999 and 2023, with an average AAMR of 0.52 and the greatest value in 2023 at 1.36 (95% CI 1.20–1.52). The greatest rise was observed between 2007 and 2023 with an APC of 13.03* (95% CI 11.77–15.09, *p* < 0.000001). In the elderly, 12,918 deaths occurred, with an average AAMR of 11.28, peaking at 24.95 (95% CI 23.63–26.26) in 2023, and showing the steepest rise between 2013 and 2023 with an APC of 12.12* (95% CI 10.55–16.96, *p* < 0.000001). Surprisingly, the trends revealed that the elderly (AAPC: 3.34% (95% CI 2.81–4.13, *p* < 0.000001) saw an overall slower increase in mortality rates compared to adults (AAPC: 6.24% (95% CI 5.31–7.65, *p* < 0.000001) (Supplemental Tables S3, S6 and S7). The proportion of deaths occurring in adults aged 65 years and older declined from 88.2% in 1999–2003 to 81.5% in 2019–2023 (Supplemental Table S1), reflecting a shift toward earlier mortality relative to the total cohort.

### 3.5. Geographical Regions (Census and States)

Among the four regions the highest number of casualties was reported in the South (5646 deaths) followed by the West (3988 deaths), Midwest (3131 deaths) and Northeast (2866 deaths) from 1999 to 2023. The highest average AAMR per million was recorded in the West at 3.05 while the Midwest region had the lowest, recorded at 2.40 in 1999–2023 (Figure 3C). All the regions experienced an increase in mortality trends from 1999 to 2023, with the West showing the greatest rise (AAPC 5.46% (95% CI 4.93–6.11, *p* < 0.000001), followed by the South (AAPC 3.72% (95% CI 3.13–4.60, *p* < 0.000001), Northeast (AAPC 3.37% (95% CI 2.72–4.19, *p* < 0.000001) and Midwest (AAPC 2.57% (95% CI 1.75–3.60, *p* < 0.000001) (Supplemental Tables S3, S8 and S9).

From 1999 to 2023, the highest number of deaths related to the conditions were reported in California, with 2116 deaths, while the lowest were seen in Idaho, with 58. Nevada experienced the greatest average AAMR at 8.27 while the lowest was in Minnesota at 2.49. The average AAMR for all the states in the years 1999–2023 was 4.00 per million. The states in the upper 90th percentile were Nevada, Washington, Mississippi, Tennessee and Oregon. The states below the 10th percentile were Florida, Virginia, Alabama, Colorado and Minnesota (Supplemental Table S10).

### 3.6. Urbanization

In the years 1999–2020, rural areas (2.54) had a higher average AAMR compared to urban areas (2.18), with both peaking in 2020 with an AAMR of 5.12 and 4.04 (Figure 3D). Rural areas recorded the greatest increase in AAMR between 2008 and 2020 at 10.96% (95% CI 8.97–14.20), while the urban areas showed their highest surge in the years between 2013 and 2020 at 12.89% (95% CI 10.96–18.28). Both rural and urban areas had an overall increase in mortality trends from 1999 to 2020 with AAPCs of 2.48% (95% CI 1.54–3.68, *p* < 0.000001) and 2.76% (95% CI 2.24–3.41, *p* < 0.000001), respectively. Urban areas experienced more deaths (8898) than rural areas (2206) (Supplemental Tables S3 and S11).

### 3.7. Place of Death

Database trends from 1999 to 2023 showed that medical facility —inpatients had a total of 13,068 deaths, with the highest number of deaths in 2023 (1518) and the lowest in 2006 (225). It was followed by nursing home/long-term care patients (725 deaths), those living in a decedent’s home (708 deaths) and medical facility outpatient or ER patients (638 deaths). Categories like Medical Facility—Dead on Arrival, Medical Facility—Status Unknown, Hospice Facility, Other and Place of Death Unknown reported suppressed data or negligible deaths across the years (Supplemental Table S12).

### 3.8. Forecast

**Figure 4:**
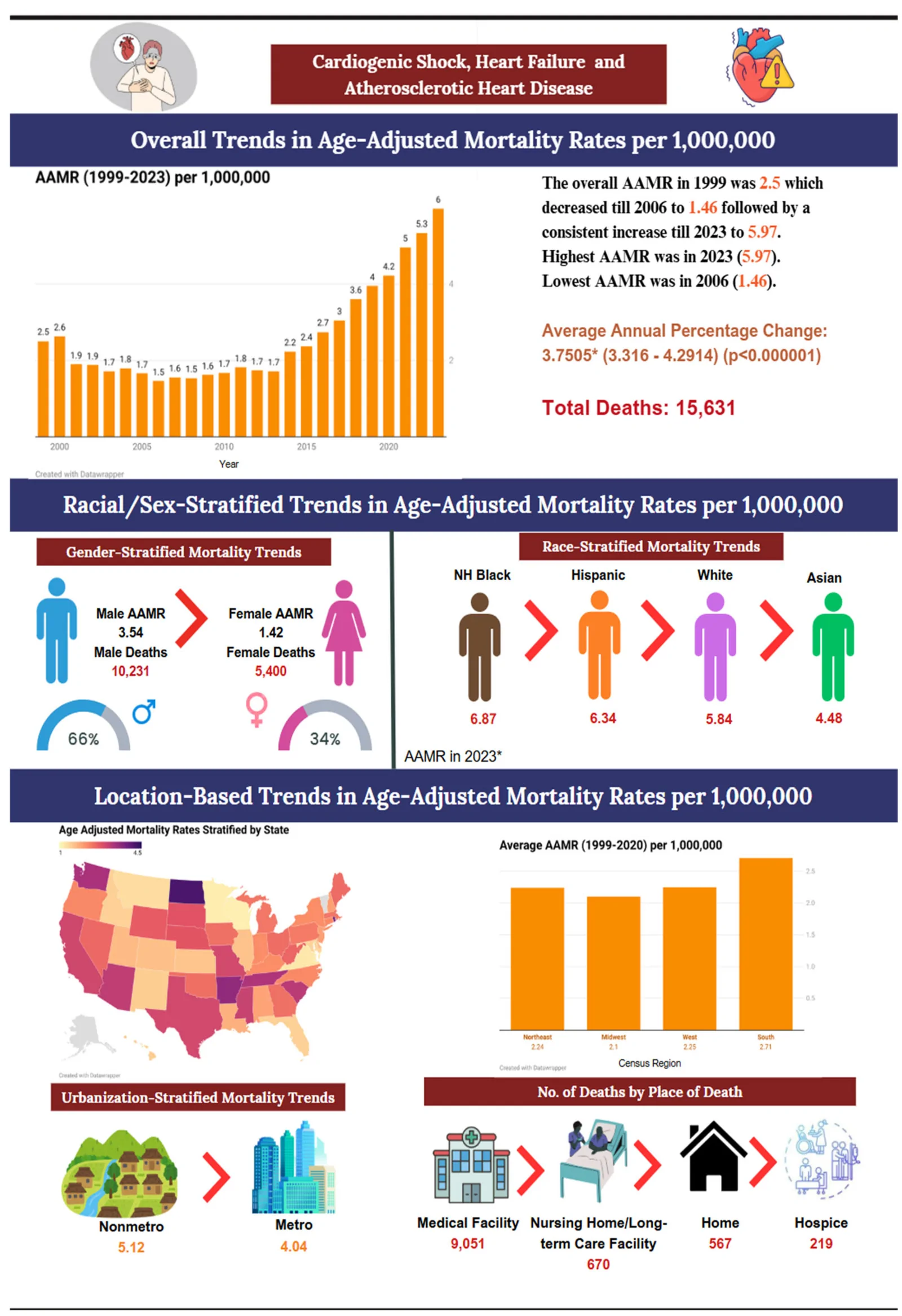
Central Illustration: Demographic profiles for cardiogenic shock, heart failure and atherosclerotic heart disease-related mortality among adults aged 25+ years in the United States, from 1999 to 2023.

Forecasting database trends till 2035 reveals rising mortality trends, with overall AAMR peaking at 11.28 in 2035 (0.32–22.25). Trends forecasted based on gender show a similar trend and significant rise in AAMRs peaking in 2035 to 16.33 per million (95% CI −39.12–71.78) for males and 4.49 (95% CI −1.02–10.01) in females. Overall mortality trends increased significantly till 2035 with an AAPC of 5.65 (95% CI: 5.58–5.70; *p* < 0.01). Mortality trends in males increased significantly during the same period, with an AAPC of 5.30 (95% CI: 5.24–5.35; *p* < 0.01), while females demonstrated a slower upward trend with an AAPC of 4.36 (95% CI: 3.88–4.69; *p* < 0.01). The demographic patterns of cardiogenic shock, heart failure, and atherosclerotic heart disease-related mortality are summarized in the central illustration (Figure 4).

## 4. Discussion

Cardiovascular disorders (CVDs) continue to rank among the leading causes of death worldwide. In many high-income countries, age-adjusted mortality rates from cardiovascular disease have decreased. However, because of population expansion and age, the absolute number of CVD fatalities may not have decreased (or may have even risen) despite falling rates [6]. In our study, the age-adjusted mortality rate increased from 2.50 deaths per million in 1999 to 5.97 deaths per million in 2023, with a marked increase after 2013. This result stands in contrast to previous declining patterns seen until the early 2000s, when significant decreases in cardiovascular fatalities were brought about by improvements in acute coronary treatment, risk factor management, and public health initiatives [7]. The reversal of these trends indicates that improvements in chronic and advanced cardiac disorders, such as heart failure and cardiogenic shock, have not kept up with the advancements made in ischemic heart disease. After 2013, the drop in cardiovascular mortality rose after 2 years of decline, which is consistent with national statistics that indicate the mortality rate from heart disease stopped declining and started to rise again at about the same period [8].

Early declines in the 1990s and 2000s were mostly caused by increased use of statins and antiplatelet medication [9], better control of hypertension and cholesterol [10], and the adoption of percutaneous coronary intervention [11,12]. The ensuing slowdown might be the result of a saturation effect [13], where it became more challenging to reduce traditional risk factors further as obesity [14], diabetes [15], and kidney disease became more common [16]. In the United States, the prevalence of diabetes more than doubled during this time [17], while the prevalence of adult obesity increased from 22.9% in 1988–1994 to over 40% by 2020 [18]. These metabolic disorders encourage endothelial damage, inflammation, and fibrosis, which directly contribute to the pathophysiology of atherosclerotic disease and heart failure [19].

The observed changes in clinical practice and coding trends in 2013 are consistent with the rise in mortality rates [15]. The 2013 American College of Cardiology/American Heart Association (ACC/AHA) guidelines redesigned the stages of management and added new diagnostic criteria for heart failure, emphasizing comorbidity control and early identification [20]. Although the changes made it easier to identify cases, it is possible that they also led to more deaths with heart failure or cardiogenic shock listed as the primary reason [21]. Furthermore, more patients lived long enough to develop chronic heart failure as survival following myocardial infarction improved because of prompt revascularization and high-intensity statins [22]. The increase in heart failure mortality in recent years can be partially explained by this transition from acute ischemia to chronic decompensated disease.

In contrast to discrete temporal changes, gender, race, age, and geographical differences in cardiovascular mortality continue to represent biological and systemic effects. An earlier onset of ischemic disease [23], increased exposure to modifiable risks like smoking and diabetes [24], and poorer adherence to long-term preventive care and medication are all factors that contribute to the higher mortality rate seen in men [25]. Although women are more likely to have preserved ejection fraction and develop heart failure later in life [26], their outcomes are influenced by delayed diagnosis and less frequent use of advanced medications or invasive evaluation [24,26,27]. These differences are further exacerbated by the fact that women are underrepresented in significant cardiovascular studies [28], which keeps the body of evidence supporting sex-specific treatment approaches limited [29]. Social and structural determinants of health also impact differences by race and ethnicity. The exposure to cardiometabolic risk is higher for non-Hispanic Black individuals [30], who are also more likely to face fragmented follow-up, delayed diagnosis, and restricted access to specialty treatment [31]. This group continues to have higher rates of hypertension, diabetes, and chronic renal disease, and poor outcomes are exacerbated by socioeconomic hurdles and drug nonadherence [32]. The intermediate death rates among Hispanic and Native American populations reflect differences in regional insurance coverage and resource allocation [33]. Rather than reflecting resilience, the pattern of age-related variation most likely reflects changes in exposure and adaptation; improved risk factor control [10], secondary prevention [7], and device-based therapy [34] have benefited older patients. However, complex comorbidities including obesity [35], metabolic liver disease [36], and chronic kidney impairment [37] are becoming more common in younger patients, which hastens heart failure [38]. Geographically, the persistence of a “heart failure belt” in the South and increased mortality in some areas of the West highlights the long-lasting relationship between socioeconomic status [39], healthcare access, and cardiovascular outcomes [3]. Distance from tertiary centers and unequal resource distribution continue to influence survival despite modest advancements in telemedicine and regional care coordination [40], highlighting that cardiovascular mortality disparities in the US are still firmly anchored in both biology and social context.

Cardiovascular mortality is still shaped by the degree of comorbidity. Chronic renal disease [41], diabetes [42], and obesity continue to be main contributors [43], while the increasing prevalence of liver disease adds another dimension [44]. It is related to cirrhosis and metabolic dysfunction-associated steatotic liver disease, indicating reciprocal organ damage [45]. Hepatic congestion and fibrosis can result from heart failure [46], while systemic inflammation and vascular damage are exacerbated by liver dysfunction, a condition known as cardio-hepatic syndrome [47]. The overlap between these diseases has become increasingly evident in epidemiological studies and clinical registries [48].

The Million Hearts Initiative and the Affordable Care Act were two policy projects that sought to increase access and stop one million cardiovascular events by 2022 [49]. National mortality patterns show that the recent increases in cardiovascular fatalities have not been entirely reversed, despite improvements in insurance coverage and the expansion of preventative care [50]. To maintain gains, broader, multi-sector strategies that tackle social determinants, physical inactivity, nutrition, and equitable care delivery will be required [51].

## 5. Strengths

This study has several strengths. It makes use of extensive, nationally representative data collected over an extended period of observation, enabling a thorough evaluation of geographic and demographic changes. The use of Joinpoint regression to identify inflection points, rather than a single linear trend, allowed us to isolate the 2013 shift from earlier and avoid averaging over the three distinct trend periods. Stratification by sex, race/ethnicity, age group, census region, urbanization, and place of death provided a granular view of disparities that has not previously been characterized for this specific combination of cardiogenic shock, heart failure, and atherosclerotic heart disease. Finally, this is, to our knowledge, the first study to examine mortality involving all three conditions together, rather than each in isolation, and to extend observed trends through ARIMA-based forecasting to 2035, offering a longer planning horizon for clinicians and policymakers than a purely retrospective analysis.

## 6. Limitations

Firstly, reliance on death certificate data raises the risk of contributing conditions being underreported and misclassified. Second, the Multiple Cause of Death file does not permit patient-level linkage, so mortality trends cannot be attributed to specific treatments, comorbidity severity, or care pathways. Third, CDC WONDER suppresses cells representing fewer than 10 deaths; this limited stratified analysis for non-Hispanic American Indian/Alaska Native, Asian/Pacific Islander, and Hispanic populations, and excluded several low-population states from state-level analysis (Supplemental Table S9). Fourth, urbanization-stratified data were only available through 2020 at the time of extraction, limiting comparability with the 2023 endpoint used elsewhere. Fifth, the final four years of the study period overlap the COVID-19 pandemic and while the upward inflection in mortality began in 2013, well before the pandemic, the acceleration observed in 2020–2021 may be partly attributable to COVID-19-related excess mortality and health care disruption. Sixth, changes in heart failure diagnostic and documentation practice following the 2013 ACC/AHA guideline update may have contributed to the observed rise independent of any true change in incidence. Finally, ARIMA forecasts to 2035 assume no structural change in risk factors, treatment, or coding practice and should be interpreted as an extrapolation of current trends.

Notwithstanding these drawbacks, the trends that have been noted are in line with those of other national and international research and offer important new information about how cardiovascular mortality is evolving in the United States.

## Data Availability

The data used in this study are publicly available from the CDC WONDER (Wide-ranging Online Data for Epidemiologic Research) database at https://wonder.cdc.gov.

## Supplementary Materials

The following supporting information can be downloaded at: https://www.mdpi.com/article/10.3390/biomedicines14081847/s1. Table S1: CS, HF and AHD-Related Deaths Stratified by Age Group (25–64 vs. ≥65 years) by 5-Year Period, United States, 1999–2023; Table S2: Number of CS, HF and AHD-Related Deaths, Stratified by Sex and Race in Adults in the United States 1999–2023; Table S3: Annual Percent Change (APC) and Average Annual Percent Change (AAPC) of CS, HF and AHD-related Age-Adjusted Mortality Rates per 1,000,000 in Adults in the United States 1999–2023; Table S4: Overall and sex-specific CS, HF and AHD–related Age-Adjusted Mortality Rates per 1,000,000 in Adults in the United States 1999–2023; Table S5: CS, HF and AHD-related Age-Adjusted Mortality Rates per 1,000,000, Stratified by Race in Adults in the United States 1999–2023; Table S6: Number of CS, HF and AHD-Related Deaths, Stratified by Age Group in Adults in the United States 1999–2023; Table S7: CS, HF and AHD -related Age-Adjusted Mortality Rates per 1,000,000, Stratified by Age group in Adults in the United States 1999–2023; Table S8: Number of CS, HF and AHD -Related Deaths, Stratified by Census Region and Urbanization in Adults in the United States 1999–2023.; Table S9: CS, HF and AHD -related Age-Adjusted Mortality Rate per 1,000,000 Stratified by Census Region in Adults in the United States 1999–2023; Table S10: CS, HF and AHD related Age Adjusted Mortality Rates per 1,000,000 Stratified by States among Adults in the United States 1999–2023; Table S11: CS, HF and AHD-related Age-Adjusted Mortality Rates per 1,000,000 in the Rural and Urban areas in Adults in the United States 1999–2020; Table S12: CS, HF and AHD related Mortality, Stratified by Place of Death in Adults in the United States 1999–2023.
