# Supplementary material for: Fatal Cardiogenic Shock in Patients with Heart Failure: National Mortality Trends, Forecasting, and the Influence of Atherosclerotic Cardiovascular Disease in the United States in 1999–2023
Source: Biomedicines. 2026 Aug 17;14(8):1847. doi: 10.3390/biomedicines14081847 (PMC13509358; doi:10.3390/biomedicines14081847)
Supplement: Supplementary file 1 [file biomedicines-14-01847-s001.zip › biomedicines-4449342-supplementary.pdf]

Supplementary Data

Supplemental Table S1. CS, HF and AHD-Related Deaths Stratified by Age Group (25–64 vs. ≥65 years) by 5-Year Period, United States, 1999–2023.

Period	Deaths, Age 25-64	Deaths, Age ≥65	% of Deaths Age ≥65
**1999-2003**	228	1,699	88.2%
**2004-2008**	218	1,364	86.2%
**2009-2013**	327	1,556	82.6%
**2014-2018**	695	2,820	80.2%
**2019-2023**	1,245	5,479	81.5%

Supplemental Table S2 Number of CS, HF and AHD-Related Deaths, Stratified by Sex and Race in Adults in the United States 1999-2023.

	Deaths					
**Year**	**Overall**	**Women**	**Men**	**NH Black or African American**	**NH White**	**Population**
**1999**	442	206	236	29	386	180408769
**2000**	469	203	266	31	404	181984640
**2001**	346	148	198	24	296	184305128
**2002**	348	159	189	22	307	186208028
**2003**	322	143	179	21	276	188090429
**2004**	340	156	184	25	287	190205384
**2005**	323	130	193	18	272	192551384
**2006**	288	118	170	29	240	195019359
**2007**	311	129	182	25	250	197403777
**2008**	320	132	188	36	253	199795090
**2009**	346	133	213	27	279	202107016
**2010**	358	143	215	34	282	203891983
**2011**	394	137	257	42	309	206592936
**2012**	391	122	269	36	314	208826037
**2013**	394	142	252	45	308	211085314
**2014**	535	190	345	58	405	213809280
**2015**	576	196	380	58	436	216553817
**2016**	687	218	469	92	507	218641417
**2017**	774	226	548	88	578	221447331
**2018**	943	287	656	123	686	223311190
**2019**	1060	326	734	145	767	224981167
**2020**	1137	355	782	127	832	226635013
**2021**	1338	418	920	175	948	228238412
**2022**	1486	449	1037	200	1038	229508599
**2023**	1703	534	1169	198	1218	231529762
**Total**	15631	5400	10231	1708	11878	5163131262

Supplemental Table S3 Annual Percent Change (APC) and Average Annual Percent Change (AAPC) of CS, HF and AHD -related Age-Adjusted Mortality Rates per 1,000,000 in Adults in the United States 1999-2023

Year Interval	APC (95% CI)	Year Interval	AAPC (95% CI)
**Overall**			
1999-2006	-7.5569* (-13.1511--5.2331)	1999-2023	3.7505* (3.316-4.2914)
2006-2013	3.9436 (-1.4787-8.9104)		
2013-2023	12.3327* (11.273-14.4627)		
**Men**			
1999-2006	-7.3604* (-14.8914--4.2441)	1999-2023	4.1622* (3.6085-4.8832)
2006-2013	5.0279 (-2.2276-10.2143)		
2013-2023	12.4174* (11.1677-15.851)		
**Women**			
1999-2006	-7.7258* (-14.9064--5.1296)	1999-2023	2.5472* (2.0707-3.108)
2006-2012	-0.3353 (-4.7161-10.9137)		
2012-2023	11.3912* (10.0867-14.076)		
**NH Black or African American**			
1999-2009	-2.0445 (-24.8049-4.5049)	1999-2023	6.1496* (4.6874-8.4462)
2009-2023	12.4189* (10.2997-18.6483)		
**NH White**			
1999-2006	-7.8496* (-10.552--6.0802)	1999-2023	3.4323* (3.0921-3.8105)
2006-2013	3.4411* (0.0396-6.7857)		
2013-2023	12.1350* (11.243-13.4166)		
**Census Region 1 – Northeast**			
1999-2010	-4.1856* (-7.4301--1.9545)	1999-2023	3.3671* (2.715-4.194)
2010-2023	10.2210* (8.8271-12.3259)		
**Census Region 2 – Midwest**			

1999-2004	-12.0894* (-26.0835--5.408)	1999-2023	2.5715* (1.7527-3.5988)
2004-2013	1.4113 (-3.6444-8.7647)		
2013-2023	11.9349* (9.6271-17.3823)		
**Census Region 3 – South**			
1999-2008	-8.7378* (-12.6435--5.814)	1999-2023	3.7278* (3.131-4.6099)
2008-2023	12.0102* (10.9984-13.6005)		
**Census Region 4 – West**			
1999-2013	0.0032 (-1.5393-1.437)	1999-2023	5.4572* (4.9364-6.1173)
2013-2023	13.5961* (12.1036-15.605)		
**Urban**			
1999-2006	-6.8140* (-13.137--4.4303)	1999-2020	2.7676* (2.2393-3.4139)
2006-2013	3.1723 (-1.6476-8.5274)		
2013-2020	12.8898* (10.959-18.2764)		
**Rural**			
1999-2008	-7.8238* (-12.9825--4.3167)	1999-2020	2.4837* (1.5362-3.6817)
2008-2020	10.9639* (8.9793-14.2063)		
**Age group (25-64)**			
1999-2007	-6.1329* (-14.8771--0.9873)	1999-2023	6.2451* (5.3111-7.6481)
2007-2023	13.0334* (11.7681-15.0895)		
**Age group (65+)**			
1999-2006	-7.4839* (-16.8616--3.9662)	1999-2023	3.3470* (2.8127-4.1318)
2006-2013	2.7574 (-3.6104-10.1728)		
2013-2023	12.1227* (10.5494-16.9606)		

*=indicates statistically significant value ($p < 0.05$)

Supplemental Table S4 Overall and sex-specific CS, HF and AHD –related Age-Adjusted Mortality Rates per 1,000,000 in Adults in the United States 1999-2023

Age-Adjusted Rate (95% CI)			
Year	**Men**	**Women**	**Overall**
**1999**	3.47 (3.01-3.92)	1.88 (1.62-2.14)	2.5 (2.26-2.73)
**2000**	3.88 (3.4-4.35)	1.82 (1.57-2.07)	2.63 (2.39-2.87)
**2001**	2.79 (2.39-3.19)	1.3 (1.09-1.51)	1.9 (1.7-2.11)
**2002**	2.63 (2.25-3.01)	1.41 (1.19-1.63)	1.88 (1.68-2.08)
**2003**	2.37 (2.02-2.73)	1.23 (1.03-1.44)	1.71 (1.53-1.9)
**2004**	2.45 (2.09-2.81)	1.36 (1.14-1.58)	1.79 (1.6-1.98)
**2005**	2.56 (2.19-2.93)	1.08 (0.89-1.27)	1.66 (1.48-1.84)
**2006**	2.12 (1.8-2.45)	0.98 (0.8-1.16)	1.46 (1.29-1.63)
**2007**	2.24 (1.91-2.57)	1.06 (0.88-1.24)	1.55 (1.38-1.72)
**2008**	2.18 (1.86-2.49)	1.07 (0.89-1.26)	1.53 (1.36-1.7)
**2009**	2.47 (2.13-2.81)	1.05 (0.87-1.23)	1.62 (1.45-1.79)
**2010**	2.49 (2.16-2.83)	1.12 (0.94-1.31)	1.67 (1.5-1.85)
**2011**	2.79 (2.44-3.14)	1.04 (0.86-1.22)	1.82 (1.64-2)
**2012**	2.82 (2.48-3.16)	0.93 (0.76-1.1)	1.74 (1.56-1.91)
**2013**	2.53 (2.21-2.85)	1.08 (0.9-1.26)	1.71 (1.54-1.89)
**2014**	3.35 (2.99-3.71)	1.38 (1.18-1.58)	2.23 (2.03-2.42)
**2015**	3.6 (3.23-3.97)	1.43 (1.23-1.63)	2.37 (2.17-2.56)
**2016**	4.27 (3.88-4.67)	1.53 (1.32-1.74)	2.74 (2.53-2.94)
**2017**	4.92 (4.5-5.34)	1.58 (1.37-1.78)	3.04 (2.82-3.26)
**2018**	5.68 (5.24-6.12)	1.95 (1.72-2.18)	3.6 (3.37-3.83)
**2019**	6.26 (5.8-6.72)	2.16 (1.92-2.4)	3.95 (3.71-4.19)
**2020**	6.53 (6.07-7)	2.35 (2.1-2.6)	4.22 (3.97-4.47)
**2021**	7.66 (7.15-8.17)	2.85 (2.57-3.13)	4.96 (4.69-5.23)
**2022**	8.39 (7.87-8.91)	2.88 (2.61-3.15)	5.33 (5.05-5.6)
**2023**	9.26 (8.71-9.8)	3.39 (3.1-3.68)	5.97 (5.68-6.26)

Supplemental Table S5 CS, HF and AHD -related Age-Adjusted Mortality Rates per 1,000,000, Stratified by Race in Adults in the United States 1999-2023

**Year**	**NH Black or African American**	**NH White**
**1999**	2.05 (1.37-2.94)	2.59 (2.33-2.85)
**2000**	2.1 (1.41-2.99)	2.67 (2.41-2.93)
**2001**	1.62 (1.03-2.43)	1.94 (1.72-2.17)
**2002**	1.44 (0.9-2.18)	1.99 (1.76-2.21)
**2003**	1.29 (0.79-1.99)	1.76 (1.55-1.96)
**2004**	1.62 (1.04-2.41)	1.82 (1.61-2.03)
**2005**	1.21 (0.65-1.77)	1.69 (1.49-1.89)
**2006**	1.66 (1.1-2.4)	1.5 (1.31-1.7)
**2007**	1.49 (0.95-2.21)	1.5 (1.31-1.69)
**2008**	1.9 (1.31-2.65)	1.51 (1.32-1.7)
**2009**	1.43 (0.94-2.1)	1.64 (1.45-1.83)
**2010**	1.74 (1.19-2.46)	1.66 (1.46-1.85)
**2011**	2.13 (1.52-2.91)	1.78 (1.58-1.98)
**2012**	1.79 (1.24-2.5)	1.78 (1.58-1.98)
**2013**	2.16 (1.56-2.92)	1.69 (1.5-1.88)
**2014**	2.5 (1.89-3.26)	2.18 (1.96-2.39)
**2015**	2.45 (1.84-3.2)	2.32 (2.1-2.54)
**2016**	3.71 (2.97-4.57)	2.63 (2.39-2.86)
**2017**	3.56 (2.83-4.42)	2.98 (2.73-3.22)
**2018**	4.87 (3.98-5.75)	3.45 (3.19-3.71)
**2019**	5.57 (4.64-6.5)	3.74 (3.47-4.01)
**2020**	4.78 (3.92-5.63)	4.14 (3.85-4.42)

**2021**	6.47 (5.48-7.46)	4.78 (4.47-5.09)
**2022**	7.34 (6.29-8.38)	5.04 (4.73-5.35)
**2023**	6.87 (5.88-7.85)	5.84 (5.51-6.17)

Supplemental Table S6 Number of CS, HF and AHD -Related Deaths, Stratified by Age Group in Adults in the United States 1999-2023.

	**Deaths**	
**Year**	**Age Group (25-64)**	**Age Group (65+)**
**1999**	41	401
**2000**	55	414
**2001**	44	302
**2002**	42	306
**2003**	46	276
**2004**	48	292
**2005**	34	289
**2006**	41	247
**2007**	37	274
**2008**	58	262
**2009**	61	285
**2010**	43	315
**2011**	64	330
**2012**	79	312
**2013**	80	314
**2014**	98	437
**2015**	107	469
**2016**	153	534
**2017**	158	616
**2018**	179	764
**2019**	203	857
**2020**	228	909
**2021**	250	1088
**2022**	265	1221
**2023**	299	1404
**Total**	2713	12918

Supplemental Table S7 CS, HF and AHD -related Age-Adjusted Mortality Rates per 1,000,000, Stratified by Age group in Adults in the United States 1999-2023

Year	Age Adjusted Rate (95%CI)	
	**Age group (25-64)**	**Age group (65+)**
**1999**	0.28 (0.2-0.38)	11.63 (10.49-12.76)
**2000**	0.38 (0.28-0.5)	11.88 (10.74-13.03)
**2001**	0.3 (0.21-0.4)	8.53 (7.57-9.49)
**2002**	0.25 (0.18-0.34)	8.57 (7.61-9.53)
**2003**	0.27 (0.2-0.36)	7.66 (6.76-8.57)
**2004**	0.28 (0.21-0.38)	8 (7.09-8.92)
**2005**	0.18 (0.12-0.25)	7.78 (6.88-8.67)
**2006**	0.23 (0.17-0.32)	6.52 (5.7-7.33)
**2007**	0.19 (0.13-0.26)	7.16 (6.31-8.01)
**2008**	0.27 (0.2-0.35)	6.72 (5.9-7.53)
**2009**	0.29 (0.22-0.37)	7.11 (6.28-7.94)
**2010**	0.19 (0.13-0.25)	7.8 (6.94-8.67)
**2011**	0.33 (0.25-0.42)	7.98 (7.12-8.85)
**2012**	0.4 (0.31-0.5)	7.25 (6.44-8.06)
**2013**	0.38 (0.3-0.47)	7.21 (6.41-8.02)
**2014**	0.44 (0.36-0.54)	9.57 (8.67-10.48)
**2015**	0.48 (0.39-0.58)	10.13 (9.2-11.06)
**2016**	0.68 (0.57-0.79)	11.19 (10.23-12.15)
**2017**	0.73 (0.62-0.85)	12.54 (11.54-13.54)
**2018**	0.81 (0.69-0.93)	15.08 (14-16.16)
**2019**	0.9 (0.77-1.03)	16.52 (15.4-17.63)

**2020**	1.06 (0.92-1.2)	17.21 (16.09-18.34)
**2021**	1.12 (0.97-1.26)	20.79 (19.54-22.04)
**2022**	1.24 (1.08-1.39)	22.16 (20.9-23.41)
**2023**	1.36 (1.2-1.52)	24.95 (23.63-26.26)

Supplemental Table S8 Number of CS, HF and AHD -Related Deaths, Stratified by Census Region and Urbanization in Adults in the United States 1999-2023.

	Deaths					
**Years**	**Northeast**	**Midwest**	**South**	**West**	**Rural**	**Urban**
**1999**	90	116	144	92	105	337
**2000**	103	126	167	73	109	360
**2001**	78	65	131	72	66	280
**2002**	85	87	103	73	78	270
**2003**	61	80	118	63	62	260
**2004**	65	68	123	84	68	272
**2005**	74	71	95	83	62	261
**2006**	68	60	85	75	54	234
**2007**	76	65	82	88	66	245
**2008**	69	81	88	82	61	259
**2009**	66	86	99	95	60	286
**2010**	66	78	106	108	61	297
**2011**	79	86	130	99	96	298
**2012**	79	95	118	99	77	314
**2013**	73	73	147	101	69	325
**2014**	112	112	170	141	104	431
**2015**	105	110	241	120	128	448
**2016**	113	116	278	180	143	544
**2017**	129	141	305	199	140	634
**2018**	172	170	363	238	170	773
**2019**	174	197	421	268	203	857
**2020**	188	209	426	314	224	913
**2021**	224	268	500	346	-	-
**2022**	235	265	560	426	-	-
**2023**	282	306	646	469	-	-

Total	2866	3131	5646	3988	2206	8898

Supplemental Table S9 CS, HF and AHD -related Age-Adjusted Mortality Rate per 1,000,000 Stratified by Census Region in Adults in the United States 1999-2023

Age-Adjusted Rate (95% CI)				
**Year**	**Northeast**	**Midwest**	**South**	**West**
**1999**	2.36 (1.9-2.91)	2.73 (2.23-3.23)	2.33 (1.95-2.71)	2.65 (2.14-3.25)
**2000**	2.71 (2.18-3.23)	2.96 (2.44-3.47)	2.7 (2.29-3.11)	2.08 (1.63-2.61)
**2001**	2.03 (1.6-2.53)	1.49 (1.15-1.9)	2.08 (1.72-2.43)	1.98 (1.55-2.49)
**2002**	2.19 (1.75-2.71)	2 (1.6-2.46)	1.58 (1.27-1.88)	1.98 (1.55-2.49)
**2003**	1.56 (1.19-2)	1.84 (1.46-2.29)	1.8 (1.48-2.13)	1.67 (1.28-2.13)
**2004**	1.64 (1.27-2.09)	1.52 (1.18-1.93)	1.83 (1.5-2.15)	2.17 (1.73-2.69)
**2005**	1.86 (1.46-2.34)	1.54 (1.2-1.94)	1.39 (1.12-1.7)	2.08 (1.65-2.58)
**2006**	1.69 (1.31-2.15)	1.31 (1-1.68)	1.22 (0.97-1.51)	1.81 (1.42-2.27)
**2007**	1.86 (1.46-2.33)	1.39 (1.07-1.78)	1.13 (0.9-1.41)	2.13 (1.7-2.62)
**2008**	1.64 (1.28-2.08)	1.71 (1.36-2.13)	1.16 (0.93-1.43)	1.89 (1.5-2.35)
**2009**	1.56 (1.2-1.99)	1.79 (1.43-2.21)	1.3 (1.06-1.59)	2.06 (1.66-2.52)
**2010**	1.56 (1.21-1.99)	1.62 (1.28-2.02)	1.39 (1.13-1.66)	2.44 (1.98-2.9)
**2011**	1.85 (1.46-2.32)	1.72 (1.38-2.13)	1.63 (1.35-1.91)	2.15 (1.74-2.62)
**2012**	1.81 (1.43-2.26)	1.85 (1.5-2.27)	1.47 (1.2-1.73)	1.98 (1.61-2.41)
**2013**	1.66 (1.3-2.1)	1.4 (1.09-1.77)	1.75 (1.46-2.03)	2 (1.6-2.4)
**2014**	2.44 (1.98-2.89)	2.15 (1.75-2.55)	1.95 (1.65-2.25)	2.71 (2.26-3.16)
**2015**	2.33 (1.88-2.78)	2.02 (1.63-2.4)	2.7 (2.36-3.05)	2.24 (1.83-2.64)
**2016**	2.39 (1.94-2.83)	2.09 (1.7-2.47)	2.95 (2.6-3.3)	3.3 (2.81-3.79)
**2017**	2.69 (2.22-3.16)	2.58 (2.14-3.01)	3.17 (2.81-3.53)	3.51 (3.02-4.01)
**2018**	3.61 (3.06-4.15)	3 (2.54-3.46)	3.65 (3.27-4.03)	4.06 (3.54-4.58)
**2019**	3.55 (3.02-4.09)	3.37 (2.9-3.85)	4.2 (3.79-4.61)	4.44 (3.9-4.97)
**2020**	3.73 (3.19-4.27)	3.61 (3.12-4.11)	4.15 (3.75-4.55)	5.21 (4.62-5.79)
**2021**	4.51 (3.91-5.11)	4.77 (4.19-5.35)	4.89 (4.46-5.33)	5.73 (5.12-6.34)
**2022**	4.58 (3.98-5.17)	4.51 (3.96-5.06)	5.3 (4.85-5.74)	6.77 (6.12-7.42)
**2023**	5.43 (4.79-6.07)	5.1 (4.52-5.68)	5.92 (5.46-6.38)	7.31 (6.64-7.98)

Supplemental Table S10 CS, HF and AHD related Age Adjusted Mortality Rates per 1,000,000 Stratified by States among Adults in the United States 1999-2023

State	Rank	Deaths	Age Adjusted Rate	Percentile
Nevada	1	199	8.27	97
Washington	2	521	6.61	94
Mississippi	3	199	6.025	92
Tennessee	4	470	5.72	89
Oregon	5	233	5.215	86
South Carolina	6	325	4.845	84
Arkansas	7	219	4.77	81
California	8	2116	4.75	78
Texas	9	1311	4.73	76
Iowa	10	195	4.7	73
Arizona	11	434	4.525	71
West Virginia	12	117	4.24	68
Kansas	13	121	4.175	65
Michigan	14	544	4.085	63
New York	15	984	3.93	60
Georgia	16	431	3.755	57
North Carolina	17	459	3.745	55
Missouri	18.5	359	3.685	50
Nebraska	18.5	108	3.685	50
Oklahoma	20	188	3.625	47
Maryland	21	208	3.61	44
Pennsylvania	22	727	3.605	42
Massachusetts	23	352	3.535	39
Ohio	24	620	3.51	36
Indiana	25	292	3.49	34
Idaho	26	58	3.445	31
Kentucky	27	193	3.335	28
Louisiana	28	173	3.26	26
Wisconsin	29	185	2.97	23
New Jersey	30	376	2.96	21
Connecticut	31	172	2.905	18
Illinois	32	442	2.815	15
Florida	33	853	2.795	13
Virginia	34	234	2.78	10
Alabama	35	196	2.715	7
Colorado	36	158	2.695	5
Minnesota	37	149	2.49	2

Supplemental Table S11 CS, HF and AHD -related Age-Adjusted Mortality Rates per 1,000,000 in the Rural and Urban areas in Adults in the United States 1999-2020

	**Age-Adjusted Rate (95% CI)**	
**Year**	**Urban**	**Rural**
**1999**	2.35 (2.1-2.6)	3.07 (2.48-3.66)
**2000**	2.51 (2.25-2.76)	3.18 (2.58-3.77)
**2001**	1.91 (1.68-2.13)	1.93 (1.49-2.45)
**2002**	1.8 (1.59-2.02)	2.25 (1.78-2.81)
**2003**	1.72 (1.51-1.93)	1.78 (1.36-2.28)
**2004**	1.76 (1.55-1.97)	1.95 (1.51-2.47)
**2005**	1.66 (1.46-1.86)	1.73 (1.33-2.22)
**2006**	1.45 (1.27-1.64)	1.48 (1.11-1.93)
**2007**	1.49 (1.3-1.68)	1.74 (1.35-2.22)
**2008**	1.54 (1.35-1.73)	1.63 (1.24-2.1)
**2009**	1.64 (1.45-1.83)	1.57 (1.2-2.02)
**2010**	1.71 (1.51-1.9)	1.62 (1.24-2.09)
**2011**	1.66 (1.47-1.85)	2.45 (1.98-2.99)
**2012**	1.69 (1.5-1.88)	1.95 (1.54-2.44)
**2013**	1.73 (1.53-1.92)	1.71 (1.33-2.17)
**2014**	2.19 (1.98-2.4)	2.55 (2.05-3.04)
**2015**	2.23 (2.02-2.44)	3.12 (2.58-3.67)
**2016**	2.62 (2.4-2.84)	3.4 (2.83-3.96)
**2017**	2.99 (2.76-3.23)	3.25 (2.7-3.8)
**2018**	3.53 (3.28-3.79)	3.94 (3.33-4.55)
**2019**	3.83 (3.57-4.09)	4.46 (3.84-5.08)
**2020**	4.04 (3.77-4.3)	5.12 (4.43-5.8)

Supplemental Table S12 CS, HF and AHD related Mortality, Stratified by Place of Death in Adults in the United States 1999-2023

	Deaths			
**Year**	**Medical Facility**	**Nursing Home/Long-term Care Facility**	**Hospices**	**Home**
**1999**	347	67	Missing	21
**2000**	356	69	Missing	32
**2001**	295	26	Missing	23
**2002**	281	37	Missing	22
**2003**	257	41	0	21
**2004**	283	27	Suppressed	24
**2005**	270	31	0	19
**2006**	238	30	Suppressed	17
**2007**	257	35	Suppressed	15
**2008**	272	25	0	16
**2009**	285	31	Suppressed	16
**2010**	296	20	17	19
**2011**	323	26	15	24
**2012**	312	24	16	33
**2013**	339	22	Suppressed	23
**2014**	462	28	Suppressed	30
**2015**	511	19	17	28
**2016**	607	23	12	37
**2017**	710	20	18	23
**2018**	851	21	29	34
**2019**	948	23	36	46
**2020**	1023	25	39	44
**2021**	1221	16	41	48
**2022**	1379	20	34	45
**2023**	1583	19	46	48
**Total**	13706	725	320	708
